# Identification of Divergent Protein Domains by Combining HMM-HMM Comparisons and Co-Occurrence Detection

**DOI:** 10.1371/journal.pone.0095275

**Published:** 2014-06-05

**Authors:** Amel Ghouila, Isabelle Florent, Fatma Zahra Guerfali, Nicolas Terrapon, Dhafer Laouini, Sadok Ben Yahia, Olivier Gascuel, Laurent Bréhélin

**Affiliations:** 1 Institut de Biologie Computationnelle, LIRMM, CNRS, Univ. Montpellier 2, Montpellier, France; 2 Computer Science Department, Faculty of Sciences of Tunis, Tunis, Tunisia; 3 Centre National de la Recherche Scientifique/Muséum National d'Histoire Naturelle, UMR7245 CNRS-MNHN, Molécules de Communication et Adaptation des Micro-organismes, Adaptation des Protozoaires à leur Environnent, Paris, France; 4 Institut Pasteur de Tunis, LR11IPT02, Laboratory of Transmission, Control and Immunobiology of Infections (LTCII), Tunis-Belvédère, Tunisia; 5 Université Tunis El Manar, Tunis, Tunisia; 6 Centre National de la Recherche Scientifique, Aix-Marseille Université, CNRS UMR 7257, AFMB, Marseille, France; University of Lausanne, Switzerland

## Abstract

Identification of protein domains is a key step for understanding protein function. Hidden Markov Models (HMMs) have proved to be a powerful tool for this task. The Pfam database notably provides a large collection of HMMs which are widely used for the annotation of proteins in sequenced organisms. This is done via sequence/HMM comparisons. However, this approach may lack sensitivity when searching for domains in divergent species. Recently, methods for HMM/HMM comparisons have been proposed and proved to be more sensitive than sequence/HMM approaches in certain cases. However, these approaches are usually not used for protein domain discovery at a genome scale, and the benefit that could be expected from their utilization for this problem has not been investigated. Using proteins of *P. falciparum* and *L. major* as examples, we investigate the extent to which HMM/HMM comparisons can identify new domain occurrences not already identified by sequence/HMM approaches. We show that although HMM/HMM comparisons are much more sensitive than sequence/HMM comparisons, they are not sufficiently accurate to be used as a standalone complement of sequence/HMM approaches at the genome scale. Hence, we propose to use domain co-occurrence — the general domain tendency to preferentially appear along with some favorite domains in the proteins — to improve the accuracy of the approach. We show that the combination of HMM/HMM comparisons and co-occurrence domain detection boosts protein annotations. At an estimated False Discovery Rate of 5%, it revealed 901 and 1098 new domains in *Plasmodium* and *Leishmania* proteins, respectively. Manual inspection of part of these predictions shows that it contains several domain families that were missing in the two organisms. All new domain occurrences have been integrated in the EuPathDomains database, along with the GO annotations that can be deduced.

## Introduction

**Figure 1:**
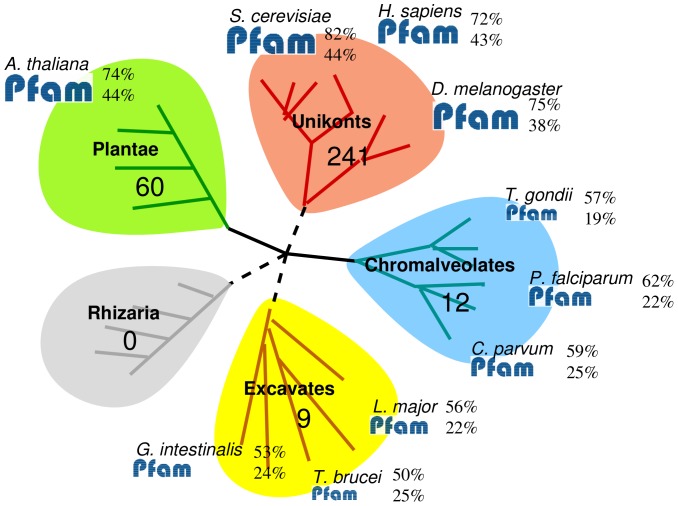
Number of sequenced genomes and domain coverage in the Eukaryote tree. This figure reports the number of genomes entirely sequenced in each of the 5 supergroups of the Eukaryote tree [58]. In each group, a few sequenced genomes are provided as example, along with statistics relative to Pfam domains (release 26): the proportion of proteins where at least one Pfam domain has been identified using recommended Pfam score thresholds (above), and the proportion of amino acids covered by a Pfam domain (below). Most of the genomes sequenced to date belong to the Unikont (241) and plant (60) super-groups. We can see that there is a marked difference in the protein domain coverage between these groups and the three other groups: while the proportion of proteins where at least one known Pfam domain is usually above 70% in Unikonts and plants, it lies between 50% and 60% in the other groups. Similarly, while the proportion of amino-acids covered by a Pfam domain is often above 40% in plants and Unikonts, it is around 22% in the other supergroups.

With the continuous improvement of genome sequencing technologies, an increasing number of new genomes are emerging everyday, enhancing basic knowledge on the diversity of organisms and providing valuable data to understand their biology and evolutionary relationships. A survey of the Uniprot database indicates, however, that this knowledge is highly unbalanced, with most of sequenced Eukaryotes being related to Plant and Unikont super-groups (see Figure 1). Since functional annotation tools have been developed based on this wealth of unbalanced data, they show limits when applied to the exploration of divergent genomes [1], [2]. This is especially true for protein domains, as illustrated in Figure.1 Domains occupy a key position among the relevant annotations that can be assigned to a protein. Protein domains are sequential and structural motifs that are found in different proteins and in different combinations and, as such, are the functional subunits of proteins above the raw amino acid level [3]. Protein domain composition provides strong clues regarding protein function. Indeed, two thirds of mono-domain proteins having the same domain also have the same function. Likewise, 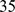
% of multi-domain proteins having one common domain present similar functions, while this rate increases to 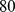
% when they share two common domains [4]. Protein domains also provide meaningful information for comparative genomics [5], [6] as well as for studying protein-protein interactions [7].

Several approaches and databases have been developed to define and identify domains. One of the most widely used domain schemes is the Pfam database [8]. The Pfam 26.0 release offers a large collection of 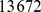 domain families. Each family in Pfam is represented by a Hidden Markov Model (HMM) of a multiple sequence alignment [9]. HMMs model both the conserved positions and gaps (insertions and deletions) of the multiple alignment [10]. HMMs are classically used as sequence/profile approaches to recognize homology and decipher family membership. When analyzing a new protein sequence, each Pfam HMM is used to compute a score measuring the similarity between the sequence and the domain using HMMER software [11]. If the score is above a given threshold provided by Pfam (each domain has its own recommended score threshold), then the presence of the domain can be asserted in the protein. However, when applied to organisms showing high evolutionary distance from the classical models which served in the construction of the HMMs, this strategy may miss several domains [12], [13]. This is the case for most eukaryotic pathogens, such as the *Leishmania* and *Plasmodium* species, where around 80% of the amino acids in proteins are not covered by any domain identified so far.

A significant improvement over sequence/profile approaches has been accomplished by profile/profile methods [14], [15]. In these approaches, profiles not only model the domain families but also the query protein sequences. It has been shown that these approaches are more sensitive and can detect remote homologues missed by sequence/profile comparisons [16]. Indeed, a profile built from an alignment of homologous proteins enables weighting of the information brought by each position of the query sequence, by distinguishing conserved from non-conserved positions [14]. HHPRED is one of the most recent profile/profile comparison approaches [16]. It enables comparison of an HMM built on a protein alignment against an HMM database like Pfam. Given a query sequence, HHPRED first generates a multiple sequence alignment (MSA) of related sequences through an iterative approach like PSI-BLAST. This MSA is then transformed into a query HMM which is compared against the HMM database. HHPRED is one of the best performing methods for fold recognition and domain boundary prediction [15]–[17]. However, although the HHPRED approach is widely used in the protein-structure prediction community to identify remote homologues with known 3D-structure, it is seldom used to annotate a whole new genome and to help predict the function of its proteins. For this task, sequence/profile comparison remains the gold standard method.

Here we use HHPRED to help annotate proteins of two main human pathogens, the kinetoplastid *Leishmania major*, that causes a cutaneous form of leishmaniasis, and the apicomplexan *Plasmodium falciparum* which is responsible for the deadliest form of human malaria. More specifically, our aim is to use HHPRED to identify new domain occurrences not already identified by HMMER using standard thresholds on these two species. We show that although HHPRED outperforms HMMER in terms of sensitivity for identifying divergent occurrences, it is not sufficiently accurate to be used as a standalone annotation tool on these particular domains. Hence, a post treatment is required to be able to distinguish between true and false positives. We propose to use domain co-occurrence property for this purpose. The co-occurrence property results from the tendency of most protein domains to preferentially appear along with few favorite domains in the same protein. This enable us to assess the occurrence of a particular domain in a protein by looking at the other domains of the same protein. We present the results achieved by this combined approach on *L. major* and *P. falciparum* species, and we show that it greatly improves the domain coverage and the functional annotations that can be attached to these organisms. Interestingly, many new domain families that had never been seen before in these organisms were discovered. We discuss these results and give a few examples that illustrate the new insights that can be deduced from these predictions and their relevance for improving the understanding of the biology of these human pathogens.

## Results

The aim of this work is to boost Pfam domain predictions using profile/profile comparison in order to enrich our knowledge on the protein domain catalogue (and hence protein functions) of the two major pathogens *L. major* and *P. falciparum*. All Pfam domains that can be identified by HMMER with the recommended score thresholds are considered as *known* in the following, and our aim is to identify new domain occurrences. Alignments were done both in global (*i.e.* the alignment extends up to the beginning and end of the Pfam HMM) and local mode (*i.e.* alignments on domain fragments are allowed). Contrary to previous HMMER versions, the last HMMER version 3.0 only handles local alignments. Hence, we used HMMER version 2 to determine the known domain occurrences in global alignment mode. Moreover, as the Pfam26 HMMs cannot be handled by HMMER2, we used the Pfam23 release instead of Pfam26 for the global mode experiments. *L. major* and *P. falciparum* protein sequences were downloaded from Trytrip (http://tritrypdb.org/) and PlasmoDB databases (http://plasmodb.org/), respectively. Each protein sequence was first transformed into an HMM by computing a multiple alignment of homologous sequences. This was done in two ways. The first approach (hereafter denoted as *phylum specific*) involves using only homologous sequences of species belonging to the same phylum as the target organism—for *P. falciparum* we use 6 *Apicomplexa*, and 6 Trypanosomatidae for *L. major* (see Methods). The second approach (*phylum non-specific*) involves using the HHBlits approach [18] on the whole Uniprot database. HHBlits proceeds in a Psiblast-like manner by iterative sequence searches (see Methods). Once all HMMs were built, they were compared to Pfam HMMs using the *hhsearch* procedure. *hhsearch* computes a score for each HMM pair [16]. This score is an adaptation of the log-odds score used for sequence/HMM comparison [11] which maximizes the co-emission probability, *i.e.* the probability that the two HMMs will emit the same sequence of residues [16]. As in most sequence similarity search programs, the significance of the score was estimated via an e-value representing the expected number of random sequences that would achieve an as high score [16]. All matches below a predetermined e-value threshold were considered for the following. Each “match” actually corresponds to an alignment between a part of the protein HMM and a part (in local mode) or the whole (in global mode) Pfam HMM. From this alignment, we first deduced the alignment between the Pfam HMM and the query protein sequence. Then, all matches overlapping a known domain on the protein sequence were removed. Similarly, when two matches overlapped, the one with the greatest e-value (*i.e.* the least likely domain) was removed. The remaining matches are hereafter denoted as *potential* domains.

### HMM/HMM comparisons do not ensure high accuracy predictions

**Figure 2:**
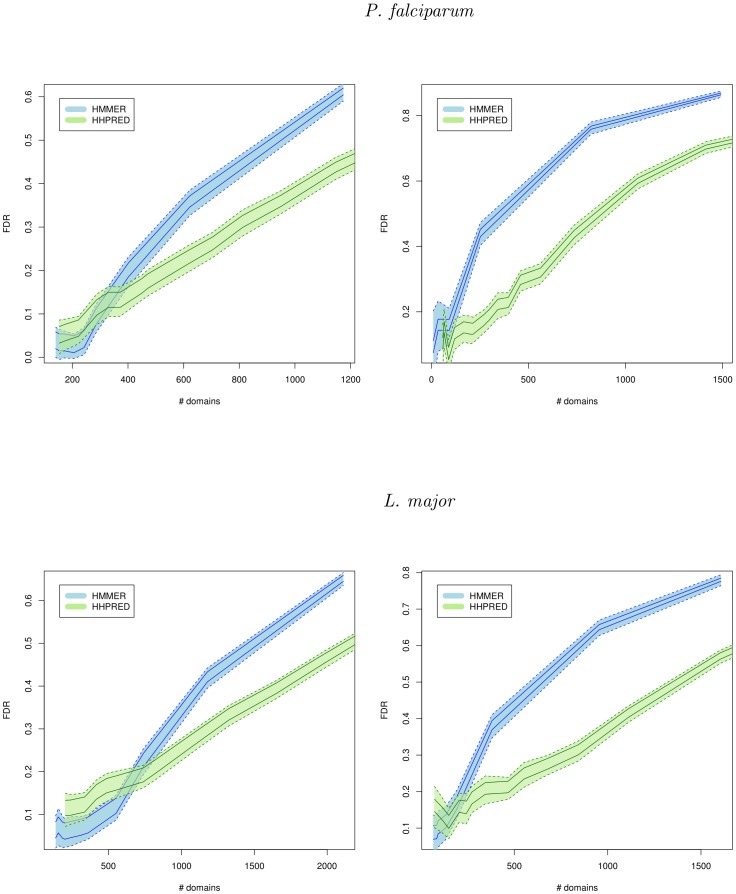
Sensitivity and accuracy of HHPRED and HMMER for *P. falciparum* and *L. major*. Number of new domains (x-axis) identified by HHPRED (green) and HMMER (blue) using local (left) and global (right) alignments for various FDRs (y-axis). For each approach, the two plain lines represent an upper and lower FDR estimate (see Methods for details). Dashed lines represent the standard error associated with these two estimates. For the sake of clarity, only the standard error above (resp. below) the upper (resp. lower) FDR estimate are represented here.

First, we wanted to estimate the overall quality of new predictions achieved by HHPRED. Several sets of potential domains of increasing size were formed using e-value thresholds ranging from 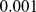 to 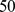. We used the procedure we proposed in N. Terrapon et al. [19] to estimate the False Discovery Rate (FDR) associated with each set of potential domains. The FDR estimation procedure is based on the well known tendency of domains to co-occur together on the same proteins [20]. A detailed description of this method is given in the Methods section. For comparison, we also ran HMMER2 (for the global mode) and HMMER3 (for the local mode) with various loose e-value thresholds. The same filtering procedure as that used for the HHPRED matches was applied to remove conflicting and overlapping matches identified by HMMER. Figure 2 reports the number of new domains identified at a given FDR by HHPRED (using the phylum non-specific approach) and HHMMER in both modes and on both species. As expected, for both approaches, when the e-value threshold increases, more domains are discovered, but the FDR associated with the predictions also increases. For low e-value thresholds, the number of new predictions is too low to provide reliable FDR estimation and it is difficult to precisely assess the two approaches based on these values. However, HHPRED does not seem to achieve accuracy below the 10% FDR threshold on the *L. major* proteome. Note that this observation concerns a very specific case: namely, all well-conserved domains have been already identified, and the method is challenged on the difficult cases only. Hence, it does not imply that HHPRED lacks accuracy for the general case. Most importantly, we can see in the figure that for moderate and high e-value thresholds, the two approaches show very different results, and HHPRED detects a higher number of domains than HMMER at same FDR. Although HHPRED does not ensure high precision results, it is more sensitive than HMMER at moderate and low precision. Hence, provided that we can filter out the false positives, numerous new domain occurrences can be expected from HHPRED predictions. At the genome scale, this kind of post-treatment must be done in a fully automatic way, and we propose to use the CODD procedure [13] for this purpose.

### HMM/HMM comparisons along with co-occurrence detection achieve high sensitivity and accuracy

The CODD procedure is a computational approach which enables us to select the most likely domains among a set of potential domains, while controlling the false discovery rates associated with the predictions. Like the FDR estimation method used above, CODD relies on the tendency of the domains to appear preferentially with few other domains. The principle is as follows (see Method for details). First, from the whole set of annotated Uniprot proteins, CODD identifies domain pairs that are highly co-occurrent, *i.e.* that are observed in the same proteins a significantly higher than expected number of times. These domain pairs are stored in the list of Co-occurring Domain Pairs (CDP). Next, given a set of potential Pfam domain occurrences, CODD selects those that form, with another domain of the same protein, a pair in the CDP list. Domains selected this way are assigned as being *certified*. Three certification types can be considered. The first and most accurate one is to use already known Pfam domains of the protein to certify the presence of the potential domains. A complementary solution is to use the other known InterPro (*i.e.* non-Pfam) domains. This usually increases the number of certifications. However, because of the heterogeneity of the InterPro database, the certifications achieved this way may be of lower quality than those achieved with Pfam domains. These first two solutions certify domains solely in proteins in which at least one domain is already known. To overcome this limitation, a third solution is to certify the potential domain by another potential domain of the protein. With this solution, all pairs of potential domains of the protein are enumerated, and the two domains are certified if the pair belongs to the CDP list. Finally, CODD uses a shuffling procedure to provide an estimate of the FDR associated with the certified domains [13]. Namely, CODD randomly shuffles potential domains of all proteins and applies the same certification process on these random domains. The number of certifications achieved on random data are compared to those done on real data, and this serves as the basis of the FDR estimate (see Methods).

We first thought to assess this approach on the already known domains using a cross-validation procedure. We selected the proteins of *P. falciparum* and *L. major* where at least two Pfam domains were already known. This represents 561 and 913 proteins in *P. falciparum* and *L. major*, respectively. Then, we randomly discarded one domain of each protein. HMMER and HHPRED (with the phylum non-specific approach) were ran to detect the potential domains below a predetermined e-value threshold, and the CODD procedure was applied, using the remaining known domains of each protein for the certification. The FDR associated with the predictions was estimated, and we computed the number of discarded domains that are recovered as well as the number of new domains that are discovered. Table 1 reports the results achieved at 3% FDR. For *P. falciparum*, around 98% of the domains predicted by HMMER+CODD belong to the discarded known-domains, and 437 out of the 561 discarded domains (78%) are recovered. For HHPRED+CODD, the results are very different: 94% of the discarded domains are recovered, while 300 certified domains are completely new. To get a rough idea about the number of false positives in both approaches, we computed the number of new domains that overlap a discarded domain. For HMMER+CODD this number equals zero, which was expected because all known domains (and hence the discarded ones) were identified with HMMER. For HHPRED+CODD, this number equal 2, which represents 0.6% of the 300 new domains, and 0.2% of the total number of certified domains, *i.e.* far less than the 3% estimated FDR. Similar results are achieved on *L. major*.

**Table 1:** Cross-validation experiments on *P. falciparum* and *L. major*.

# total certif.			# recovered domains	# overlaps
*P. falciparum (561)*	HMMER+CODD	448	437 (78%)	0
	HHPRED+CODD	828	528 (94%)	2
*L. major (913)*	HMMER+CODD	679	679 (74%)	0
	HHPRED+CODD	1345	838 (92%)	7

The test was done on the 561 and 913 proteins of *P. falciparum* and *L. major* that have at least two known Pfam domains, respectively. The table reports the number of domains identified by HMMER and HHPRED that are certified by CODD at 3% FDR. Columns “# total certif.” and “# recovered domains” reports the total number of certified domains and the number of discarded domains that are recovered, respectively. Column “# overlaps” reports the number of newly certified domains that overlap a discarded domain.

**Figure 3:**
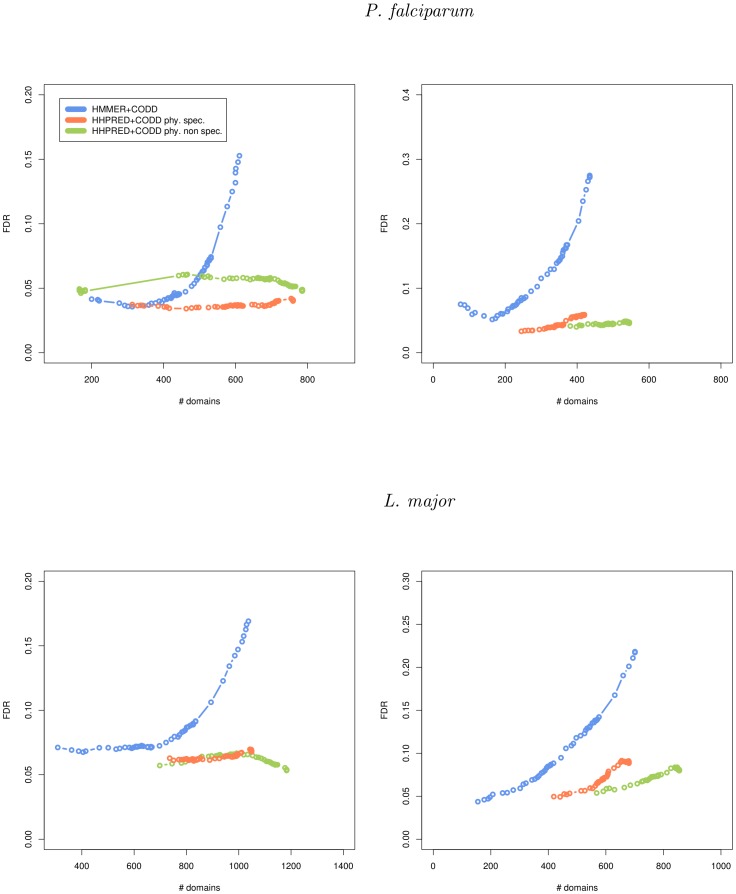
Sensitivity and accuracy of HHPRED+CODD and HMMER+CODD using the known Pfam domain occurrences for certifications. This figure reports the number of new domains (x-axis) certified by HHPRED+CODD (in orange and green for the phylum specific and non-specific approaches, respectively) and HMMER+CODD (blue) using local (left) and global (right) alignments for various FDR thresholds (y-axis).

We then ran the CODD procedure on all potential domains detected by HHPRED—with both the phylum specific and non-specific approaches—using already known Pfam domains for the certifications. This was done in the local and global alignment modes of HHPRED. Figure 3 summarizes the results achieved on *L. major* and *P. falciparum* in both modes and using different e-value thresholds. For each threshold, the number of certified domains among the potential domains below this threshold was computed, and the FDR associated with these predictions was estimated. For comparison, we also included the results achieved by CODD on new domains predicted by HMMER2 (global mode) and HMMER3 (local mode) for different e-value thresholds. As we can see, and in accordance with our first test, HHPRED greatly outperforms HMMER when used in conjunction with CODD. Moreover, the CODD procedure improves the accuracy of the approach, and FDRs as low as 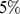 can now be achieved.

The phylum specific and non-specific approaches approaches give close results in terms of accuracy. The non-specific approach outperforms the specific one for Leishmania whereas the species-specific approach achieves better results for Plasmodium proteins. This can be explained by the fact that homologous proteins in Leishmania species usually have high sequence identity. Hence, the multiple alignments built from these sequences may lack diversity. The same trend holds for the two other types of certification (non-Pfam and potential domains, see Figures S1 and S2), except that the FDR does not always achieve the 5% threshold for these certifications.

Table 2 summarizes the results achieved at 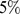 and 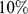 FDR for the different certification approaches on *P. falciparum* and *L. major*. Overall, 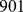 and 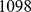 new non-redundant domains were predicted at 5% FDR on the two organisms. “Non-redundant” means here that only one occurrence of each domain family is considered for each protein—occurrences matching a domain family already known in the protein are not considered, and multiple occurrences of the same family are counted only once. In comparison with the 4423 and 6162 non-redundant known Pfam domains of these organisms, this corresponds to an increase of 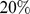 and 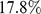 for *P. falciparum* and *L. major*, respectively. The majority (about 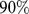) of certified domains identified by HMMER were also identified by HHPRED. Interestingly, several predicted domains had never been seen in the studied species (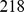 for *L. major* and 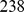 for *P. falciparum*), which corresponds to an increase of around 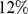 of the domain diversity for both organisms.

**Table 2:** New Pfam domains (release 26) identified at 5% and 10% FDR.

Pfam			Interpro	Pot.	All
*P. falciparum*	# dom.	621/621	−/727*	485/581	901 (20.3%)/1096 (24.7%)
	new fam.	181/181	−/214*	125/155	238 (13.4%)/304 (17.1%)
*L. major*	# dom.	1098/1098	−/123*	−/972	1098 (17.8%)/1732 (28.1%)
	new fam.	218/218	−/27*	−/140	218 (10.8%)/287 (14.2%)

The table reports the number of new domains identified by HHPRED (local mode, phylum non-specific approach) and CODD for the three certification types: known Pfam domains (Pfam), known InterPro non-Pfam domains (Interp), potential domains (Pot). “All”: results achieved when combining the 3 types. “# dom.”: number of new domains identified. “new fam.”: number of domain families that were not previously known in any protein of the organism. In each cell, the left and right numbers report the result at 5% and 10% FDR, respectively. Column “All”: The number in parenthesis reports the proportion of already known domains or family this represents. ^*^For the certifications by Interpro domains, this is the number of domains identified at 12% FDR because no FDR below 10% can be achieved by this certification type.

One issue we have eluded so far concerns the specificities of the domain combinations in species like *P. falciparum* and *L. major*. Domain pairs in the CDP list have been selected on the basis of the whole set of Uniprot proteins. Because *P. falciparum* and *L. major* likely possess specific domain-combinations, a question remains about the impact of these specificities on the certification process. First, it is important to note that a combination absent from the CDP list does not totally impede the certification of the domains involved in this combination: in proteins with more than two domains, when a domain 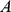 cannot be certified by a domain 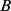 because the pair 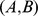 is not in the CDP, it may still be certified by a third domain. To go further in the analysis, we enumerated all domain combinations present in at least one protein of *P. falciparum* and *L. major*, and compared it to the domain combinations found in any Uniprot proteins restricted to Vertebrates, Fungi, Plants, Bacteria, and Archaea. The number of domain pairs present in *P. falciparum* and *L. major* are 976 and 1008, respectively. Among these, 96 and 159 are not found in the other phyla. We then identified the highly co-occurrent domain pairs of *P. falciparum* and *L. major* using the same procedure as the one used to select the CDP list. With a p-value of 1%, we found 834 and 922 highly co-occurrent domain pairs in *P. falciparum* and *L. major*, respectively. Among these, only 19 and 41 are missing in the original CDP list, respectively.

### GO annotations transfer

We next investigated GO annotations that could be deduced from all newly identified domains. Some domains have been associated with specific GO terms by the InterPro consortium. The policy is to associate with a given domain the annotations shared by all annotated proteins possessing this domain. This stringent policy potentially misses numerous domain-annotation associations, because a single protein possessing a domain 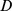 but erroneously lacking annotation 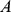 may prevent the 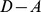 association. Hence, we chose to relax the 100% threshold. Namely, we looked for all 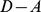 associations where at least 95% of annotated proteins with domain 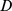 also have annotation 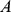. 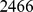 domains can be annotated in this way. Moreover, we extended the strategy to domain combinations (as described in [21]), and looked for additional GO terms that could be deduced from the combination of two domains. To this end, we enumerated all Pfam domain pairs in the proteins of Swiss-Prot, and identified, for each combination, the GO terms shared by 95% annotated proteins where the pair was present (only pairs observed in at least 5 annotated proteins were considered). We found 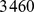 Pfam domain pairs associated with at least one specific GO annotation. All associations between domain combinations and GO terms are available in Table S1. We then investigated GO annotations that could be deduced from these association in the two species. Table 3 provides, for each species, the number of known annotations, the number of annotations that can be deduced from the known protein domains using our associations, and the number of annotations brought by the new domains (5% FDR) either solely or in combination with another (known or new) domain. In this latter category, we distinguish between annotations that were already known or that could be deduced from the already known domains, and the really new annotations brought by our domain predictions. Altogether, the new domains brought 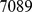 and 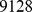 annotations for *P. falciparum* and *L. major*, respectively. Among these, 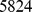 (82%) and 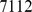 (80%) confirm already known annotations or annotations deduced from known domains, while 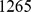 (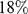) and 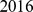 (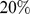) are completely new.

**Table 3:** New GO annotations at 5% FDR.

	# known GO	# GO known dom.	# GO new dom.
*P. falciparum*	*15661*	*3228*	*1265 (5824)*
*L. major*	*11958*	*6750*	*2016 (7112)*

“# known GO” is the number of known GO annotations from EuPathDB; “# GO known dom.” is the number of GO annotations that can be deduced from already known domains; “#GO new dom.” is the number of new GO annotations that can be deduced from new domains. Numbers in parenthesis report the number of annotations that confirm already known annotations or annotations deduced from known domains.

One point that is important to bear in mind is the question of the functional conservation of the divergent domains identified by our approach. It is a well known fact that the functional similarity of two homologous proteins is generally a function of their sequence similarity (see for example [22]). Hence, although the divergent domain occurrences identified by the HHPRED+CODD approach likely belong to the same functional category as the sequences used to define the domain families of Pfam, it is important to note that they may have different specific functions.

### Newly predicted domains

All new domain occurrences are available in Tables S4 and S5, and have been integrated in the EuPathDomains database (http://www.atgc-montpellier.fr/EuPathDomains/), along with the GO annotations that can be deduced from all these new domains. EuPathDomains is a protein domain database dedicated to most of the eukaryotic pathogens present from the EuPathDB portal (http://eupathdb.org/eupathdb/).

A survey of knowledge gained by the HHPRED+CODD approach can serve as a starting point for developing new hypotheses to gain further insight into the biological mechanisms of these parasites. Hence, we sought to analyze and characterize the specificities of newly discovered domains and their contribution to the understanding of parasite biological functions. We then tried to assess the functional relevance of these novel annotations based on the known and predicted properties of the corresponding protein in each parasite. For this purpose, we performed a detailed case by case manual analysis of the new domains families observed in each species. As shown in table 2, with an FDR 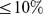, a total of 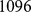 and 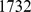 domains were identified in *P. falciparum* and *L. major* proteins by combining CODD and HHPRED in local alignment mode, respectively. For a first investigation we chose to focus our discussion on the domains identified by global alignment and that had never been observed on any protein of the studied organism, since they are likely to be the more relevant in terms of functional novelty. To further reduce these examples to a number that can be handled manually, we chose to examine only domains identified by the phylum specific approach and which are not identified by HMMER+CODD at 10% FDR. This represents a set of 36 and 37 domains in *P. falciparum* and *L. major*, respectively. In our analyses, we considered known functions of the protein on which the domain was found. Particular attention was given to the position in the protein sequence where the novel domain was discovered as well as to the description and GO annotations associated with this domain. We then tried to investigate the functional relationship with the biological function of the protein. For some of these predictions, we found direct support in the literature. We also took into consideration other species where the domain is known and tried to find common points and explanations that could help to understand the association of this domain to the protein. In the case of hypothetical proteins, this may suggest the attribution of new functions. When a predicted domain could be specific to one developmental stage, we looked at the transcriptional profiles of the protein. From all this information, we tried to infer biological knowledge that could be gained from these predictions. We discuss below several examples that have been found in the two species. Interested readers can find the full analysis in Tables S2 and S3.

#### Analysis of domains discovered in *Plasmodium* proteins

At first sight, we noticed that the majority (about 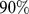) were in agreement with the global functional knowledge of the corresponding proteins, providing additional or refined features mainly consistent with already known protein domains or functions. For example, a *WH2* domain known for its interaction with *actins*, was detected in the protein *PFL0925w*, currently named “*formin 2, putative*” in PlasmoDB.

In some occurrences, the new domains (for example, *DENN* and *TFIID_90k*) do provide or precise functions to proteins in which the previously identified domains were either not very informative or had no precise biological function attached to it (*i.e. WD40*). *WD40* domains, which are among the 10 most currently found domains in eukaryotic proteins, indicate interaction properties with other proteins, peptides or nucleic acids and hence, *WD40* proteins are involved in a wide variety of functions such as *signal transduction*, *cytoskeleton assembly*, *RNA maturation*, *chromatin dynamics*, *vesicular trafficking*, etc. [23]. The possibility, offered here, to identify additional domains paired with *WD40* domains is therefore invaluable to better qualify the functions of this otherwise highly diverse *WD40* proteins family. Example for such a refinement are listed in Table S2 and are also illustrated further in the text for protein *PF3D7_1138800*.

A series of new domains putatively involved either in invasion or egress were also discovered. A “*Mar sialic bdg”* domain was indentified in *PCRMP2* (*MAL7P1.92*), that could be used by salivary gland-sporozoites to invade the insect-host tissues [24], as was found in *T. gondii* [25] or other apicomplexan parasites [26]. A *LysM* domain was identified on *PFA0130c*, a member of the “*serine/threonine protein kinase, FIKK family*” that could play an important role in erythrocyte modelling [27], [28]. Indeed, *LysM* domain are found in a variety of enzymes involved in bacterial cell wall degradation [28].

A very interesting discovery concerns a *DHQ_synthase* domain, that was predicted at position 1–171 of the *PFB0280w* protein. *DHQ* stands for 3-dehydroquinate and indeed, the enzyme encoding *3-dehydroquinate synthase activity*, which is involved in the first steps of the shikimate pathway, has yet to be identified in *Plasmodium* [29] (see also Malaria Metabolic Pathways http://sites.huji.ac.il/malaria/). The shikimate pathway allows the synthesis of aromatic amino acids and is present in plants and microorganisms. It has been shown to be active in *Plasmodium* and is considered as an attractive drug target because it is absent from mammalian cells [29], [30]. However, proteins encoding the first 4 activities of this 7-step pathway (*DHQ synthase activity* corresponds to the second step) are still elusive in *Plasmodium* [29]. *PFB0280w*, which is currently described as bi-functional enzyme encoding steps 5 and 6 of the shikimate pathway—it harbours the *EPSP* and the *SKI* domains, both involved in the shikimate pathway—, could therefore be a malarial ortholog of the *arom* protein, a penta-functional enzyme typically found in *fungi*. Interestingly, in *T. gondii*, a *DHQ-synthase* domain has also been found in a penta-functional enzyme homologous to the fungus *arom* protein.

Finally, this method could even provide functions for proteins previously totally devoid of both domains and annotations, thanks to the use of potential domains for the certification process. We predicted, for example, the *Rad21_Rec8_N* and *Rad21_Rec8* domains in the conserved *Plasmodium* protein *PF14_0380* that did not have any known GO function. Both predicted domains suggest involvement in the mediation of sister chromatid cohesion during mitosis and meiosis, as part of the cohesin complex. The indication in PlasmoDB that *PF14_0380* interacts with *PFC0155c* protein, annotated as “*DNA directed RNA pol. Subunit I*” further supports this hypothesis.

#### Analysis of domains discovered in *Leishmania* proteins

As for *P. falciparum*, several *L. major* novel domains are complementary to known protein features and confirm functions associated with them. For example, we predicted the *Ku-c* domain in the C-terminal region of the *LmjF29.1050* protein, which already possesses the *Ku-N* and *Ku70/Ku80* domains. Besides these predictions, several other domains suggest new functions for proteins with no or only very general functions. As for *P. falciparum*, a strikingly high number of domains in this case were predicted in association with the ubiquitous *WD40* domains. Among the most interesting predictions, we can cite, for example, the *Rhomboid* domain, predicted in the *LmjF24.1580* protein. *Rhomboid* domains belong to proteins of a large family of intra-membrane serine proteases. Their conservation throughout almost all branches of life suggests involvement in key biological events with various functions: triggering of signaling events in *Drosophila*, association with pathogenesis in protozoan parasites, and parasite proliferation in *T. gondii* [31]. *Rhomboid-like* proteases had been described as being localized in the secretory pathway or belonging to mitochondria. Prior to this study, no Pfam *Rhomboid* domain have been described in *L. major*, whereas partial *Rhomboid* domains have been described in other *Leishmania* species. In *LmjF24.1580*, the presence of a mitochondrial like N-terminal targeting sequence suggests a putative mitochondrial function for this protein [32]. Despite their high conservation, *Rhomboid* proteases seem to display different functions in distinct organisms, so it is unlikely that a single widespread function is conserved among all species. Examples of mitochondrial function associated with oxidative stress signaling have been described in yeast, or in Parkinson's disease in humans, whereas Rhomboid proteases conserved in extracellular pathogens have been associated with host cell invasion or, in the case of the extracellular ameba *Entamoeba histolytica*, to immune evasion [33].

### Investigation of domains involved in transcriptional regulation

Parasitic protists have often evolved transcriptional regulation mechanisms different from classical higher-eukaryotes models [34]. In trypanosomatids, this is partly due to the particular polycistronic organization of genes without a clearly identified *RNA polymerase II* (*RNAP II*) transcription system (except for *SL-RNA*). This is mainly illustrated by the absence of several *RNAP II*-related transcription factors (TF). Indeed, although several basal TFs have been identified in *Leishmania* species, several others seem to be missing in these species and other trypanosomatids [35], [36]. The picture is a little different in *P. falciparum*. While almost all proteins necessary for the basal transcription apparatus have been identified, there appears to be a lack of specific TFs [37]. With the notable exception of the *AP2* domain [38], most attempts for the identification of specific TFs in *P. falciparum* have failed. In these conditions, the discovery of *DNA binding* domains involved in transcriptional regulation may be of great interest in *P. falciparum* and *L. major*. We retrieved from the Pfam website a list of domains associated with GO terms related to transcription, and searched for occurrences of these domains in our predictions at 5% FDR (local mode, phylum non-specific approach). We discuss below the most interesting discoveries of this analysis in *P. falciparum* and *L. major*.

#### Domains discovered in *P. falciparum* proteins

We predicted, for example, a *TFIID_90kDa* domain known to be found in subunits of transcription factor *TFIID* in the protein *PF3D7_1138800* (previously *PF11_0399*, *PF11_0400* and *PF11_0401*), annotated “*conserved Plasmodium protein, unknown function*”. Currently, this very large *P. falciparum* protein predicted to be nuclear, has domain annotations solely upstream of position 1500 for a series of *WD40* domains. The available proteomics data showing tracks in diverse extracts among which nucleus is also in agreement with the proposal that this protein could be a novel subunit of *TFIID*.

The discovery of both *TFIIA_gamma_N* and *TFIIA_gamma_C* domains on the *PF3D7_0933700* protein currently annotated “*conserved Plasmodium protein, unknown function*” is of great interest. This little protein is currently totally devoid of domain annotation in PlasmoDB, although it has several GO annotations related to *DNA-dependent transcription*. Interestingly, the *TFIIA_gamma* subunit is characterized by a conserved structure, with 4 helices in the N-terminal domain and 12 beta barrels in the C-terminal domain. Such domains are indeed predicted for *PF3D7_0933700*, at the proper position, further suggesting that this protein is indeed the gamma subunit of *TFIIA* [39], [40].

We also discovered a *TFIIE_alpha* domain in the protein *PF3D7_1145800*. The general transcription factor *TFIIE* has an essential role in eukaryotic transcription initiation together with *RNA polymerase II* and other general factors. We also identified a *HTH_9* domain at the beginning of the protein. This protein is currently annotated “*conserved Plasmodium protein, unknown function*”, but PlasmoDB reports GO annotations in agreement with our observation, in particular the GO:0005673 annotation “*transcription factor TFIIE complex*”. Therefore this protein likely corresponds to the *TFIIE_alpha peptide*, as suggested by our study.

Another interesting prediction was the discovery of the *Auxin_resp* domain (*PF06507*) of the very large *P. falciparum* protein *PF14_0463*, currently annotated “*chloroquine resistance marker protein (CRMP)*”. This domain occurs in several plant transcription factors that are responsive to the *Auxin* hormone, and their conserved structure includes a N-terminal *DNA binding* domain and a C-terminal protein-protein interaction domain [41]. So far, *PF14_0463* has a single domain, *PFI12047* (*DNMT1-RFD, cytosine specific DNA methyltransferase replication foci* domain) at positions 793-943, which is also a *DNA binding* domain. Interestingly, this *Auxin_resp* domain has so far been identified in four other Apicomplexan proteins (*B9PN31_TOXGO*, *B6KF11_TOXGO*, *B9Q8D8_TOXGO*, *F0VLG9_NEOCL*). All of them also have a *DNMT1-RFD* domain upstream, highlighting structural conservation in the phylum. In addition, *PF14_0463* is currently reported to be nuclear, and to interact with a large number of proteins, including several nuclear proteins (*PFD3D7_1464000*, *PF3D7_0729400*, *PFD3D7_1212900*), which is in accordance with transcription factor activity. Note that GeneDB currently recommends that the “*chloroquine resistance marker protein*” annotation should be discontinued.

Two domains, *RNA_pol_Rpb1-3* and *RNA_pol_Rpb1-4*, were discovered in two proteins encoded next to each other by the apicoplast genome: *PFC10_API0016* and *PFC10_API0017* [42]. These two proteins are currently annotated as *rpoC* and *rpoD*, respectively. Interestingly, at present, domains *RNA_pol_Rpb1-1* and *RNA_pol_Rpb1-2* have been annotated for *PFC10_API0016*, upstream of our newly discovered *RNA_pol_Rpb1-3* domain and a *RNA_pol_Rpb1-5* domain has been annotated for *PFC10_API0017*, upstream of our newly discovered *RNA_pol_Rpb1-4* domain. This discovery is another example where our new domains confirm and further define the structure and function of an already annotated protein. Note that the two genes encode a prokaryotic-type *RNA polymerase* that is known to be split into two polypeptides in *Archae* and chloroplasts [43].

#### Domains discovered in *L. major* proteins

*LmjF.28.1740* is a hypothetical protein in which we identified a novel domain called *NusB*. *NusB* is a prokaryotic transcription factor involved in the antitermination process, *i.e.* the phenomenon whereby RNA polymerases terminate transcription at specific sites or read through terminators, which is crucial for the regulation of gene expression [44]. While this protein acts as a monomer in *Escherichia coli*, it has been described to act as a dimer in *Mycobacterium tuberculosis* [45]; the dimerization might potentially be used to maintain *NusB* in an inactive form until it is recruited for the antitermination process [46].

A novel domain called *CarD_CdnL_TRCF* has been identified in *LmjF.32.2230*. This gene is annotated as “*ATP-dependent RNA helicase putative*” and bears domains related to this helicase function. The *CarD_CdnL_TRCF* domain then adds a new function to the protein, putatively related to a repair mechanism during transcription. Indeed, *TRCF* (*Transcription-Repair-Coupling Factor*), for instance, is known to bind to *UvrA*, the *DNA damage recognition protein*, in order to increase strand repair during transcription [47]. In trypanosomatids, the necessity to maintain an efficient repair mechanism is described in particular for the kinetoplastid DNA (kDNA), which is subjected to intensive endogenous oxidative damage. The efficiency of kDNA maintenance is thus a crucial mechanism to repair oxidative damage [48].

The *LmjF.33.2810* gene is annotated as a “*transcription elongation factor-like protein*”. We were able to complement this annotation by the *Med26* domain (*PF08711*). *Med26*, or *TFIIS helical bundle-like* domain, is present in the N-terminal part of the *TFIIS* protein. This protein, also called *Med26* protein, is part of a large complex of 33 proteins called *Mediator*, largely conserved from plants to humans [49]. *Mediator* is able to link DNA transcription regulators (activators and repressors) to the pol II initiation machinery mainly through physical interaction with DNA specific signals and pol II subunits [50]. *TFIIS* seems to be a multifunctional protein acting as a transcription elongation factor involved in increasing the *RNAP II transcription* rate as well as a protein involved in controlling the early stages of the transcription cycle [51]. The *Med26* domain has also been found in *LmjF.33.2820*, a hypothetical protein bearing *TFIIS*-associated interpro non-PFAM domains. Interestingly, we also discovered a *TFIIS C* domain in this protein, which is a zinc finger motif that is also found in the *TFIIS*.

As for *P. falciparum*, our approach also allowed us to relate proteins already involved in the transcription process to novel domains associated with *RNAP II transcription*. *RPB1* is the largest subunit of pol II, constituting, through different subunits, the *DNA binding* domain of pol II. We identified the novel *RPB1_1* domain in *LmjF.16.1350*, annotated as the *DNA-directed RNA polymerase I* largest subunit and already bearing different *RPB1* domains. Additionally, *TF_Zn Ribbon* is a *zinc finger* motif found in *transcription factor IIB* (*TFIIB*), one of the subunits involved in eukaryotes in promoter recognition and interaction with pol II. This domain has been identified in the *LmjF.25.0440* protein, annotated as a *putative transcription factor*.

## Discussion

We have shown that profile/profile approaches like HHPRED can boost protein domain annotation. This is especially useful for species that have greatly diverged from the classical plant and Unikont model organisms, like most eukaryotic pathogens. Although the approach does not seem to be sufficiently accurate to be used as a standalone tool for identifying the divergent domain occurrences that have not been identified by classical sequence/profile approaches, it is much more sensitive than these latter, and actually enhances the annotations of several hundred proteins when used in combination with the co-occurrence domain discovery approach (CODD). For *P. falciparum* and *L. major*, HHPRED+CODD enabled us to discover 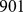 and 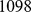 new domains at an estimated FDR of 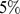 FDR, respectively.

One issue of our approach is that it applies to multi-domain proteins only. First, it is worth noting that these proteins are thought to represent a large part (around 80%) of Eukaryotic proteins [52]. Moreover, the term “domain” is used here in a very broad sense. Besides long domains, which independently folds into a particular 3D structure, the term also encompasses motifs as short as a dozen amino-acids—for example, more than 300 domain families have less than 30 amino acids in Pfam. However, it remains true that, because they are composed of a single domain, a significant number of proteins cannot be annotated by our approach. For these proteins (and the other ones as well), a solution would be to fit the Pfam HMMs to the specificities of the target proteome, and to rescan the protein sequences with these new models. A simple and efficient solution for this is to incorporate the domains occurrences already identified in the species into the Pfam seed alignment, and to train a new HMM on this alignment [19].

All predictions along with the GO annotations that can be deduced have been integrated in the EuPathDomains database, a protein domain database dedicated to eukaryotic pathogens. Close analysis of some of the predictions involving Pfam domain families that were unknown in *P. falciparum* and *L. major* showed that the approach identifies key domains that were missing to date. For example this analysis revealed one of the missing enzymes involved in the first step of the shikimate pathway, an attractive drug target in *P. falciparum* because of its absence in mammalian cells. Importantly, several predictions reveal new domains in proteins currently devoid of any domain annotation. This is for example the case for the *P. falciparum* protein *PFI630c*, which is the gamma subunit of *TFIIA* according to our analysis. Our approach is fully automatic and can be applied on any genome. Hence, it could be of great help for annotating all genomes that are phylogenetically distant from classical model organisms, and we intend to apply it to all other pathogens in EuPathDomains.

## Methods

### HHPRED predictions

We used HHPRED from the HH-suite 2.0 in our experiments. First, each query protein sequence was used to build a multiple sequence alignment (MSA). This was done using two approaches, using either only the homologous proteins in close species, or every sequenced homologue, via the HHblits method [18].

#### Phylum specific approach

For *L. major*, six species were included: four *Leishmania* species (*L. major*, *L. infantum*, *L.braziliensis* and *L. mexicana*) and two *Trypanosoma* species (*Trypanosoma cruzi* and *Trypanosoma brucei*) (http://tritrypdb.org/tritrypdb/). For *P. falciparum*, six species were analyzed: *P. falciparum*, *P. vivax*, *P. yoelii*, *P.berghei*, *P. chabaudi* and *P. knowlesi*. For each *L. major* and *P. falciparum* protein, we extracted its homologs in the set of selected closest species from the OrthoMCL database [53]. 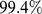 of *L. major* and 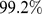 of *P. falciparum* proteins have at least one homologue in the selected species, respectively. The majority of *L. major* proteins (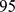
%) have orthologs in the three other *Leishmania* species. For *P. falciparum*, 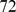
% of proteins have homologs in the five other *Plasmodium* species. When a protein has paralogs in the query species (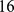
% of *P. falciparum* proteins and 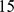
% of *L. major* proteins), these paralogs were also considered. Each query protein sequence was aligned against its homologs using Muscle [54].

#### Phylum non-specific approach (HHblits)

HHblits proceeds in a Psi-blast-like manner by iteratively aligning additional homologous sequences on the query protein. HHblits was run with default parameter values—3 iterations, local alignment mode, and 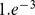 e-value threshold. The only difference is that we required a 20% minimal sequence identity threshold with the query sequence instead of the default 0%-threshold to ensure that only real homologues are included in the MSA.

All generated MSAs were then transformed into an HMM profile using the *hhmake* procedure of HHPRED. *hhmake* was run with default parameters, except for the maximum sequence identity parameter that we set at 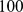 because the sequence identity is very high especially in *Leishmania* proteins. Each HMM was then compared with all HMMs of the Pfam database. We considered, for the following experiments, hits with e-values ranging from 0.1 to 10. As explained in section Results, the experiments were done using local and global alignment modes, using Pfam 26 and Pfam 23 HMMs, respectively. From each alignment of a protein-HMM and a domain-HMM, the position of the potential domain on the query sequence was deduced. Each potential domain overlapping an already known domain of the protein (*i.e.* all domain occurrences identified by HMMER below the stringent score threshold provided by Pfam) was discarded. Similarly, when two potential domains overlapped on the protein sequence, the one with the greatest e-value (*i.e.* the least likely) was removed.

### FDR estimation

Let 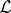 be a set of new domain occurrences identified by HMMER or HHPRED. We want to estimate the FDR associated with 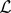, *i.e.* the probability 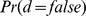 for 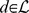. For this, we used the approach proposed in Terrapon et al. [19]. This procedure utilizes the tendency of the domain to appear preferentially with a few other favorite domains in the same proteins. The first step is to identify, from the whole set of annotated Uniprot proteins, domain pairs that are conditionally dependent, *i.e.* that are observed in the same proteins a significantly higher number of times than expected at random. This is achieved with the Fisher's exact test to cope with potentially small sample sizes. A p-value is computed for each domain pair, and pairs below a given threshold are stored in a set 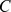 of Co-occurring Domain Pairs (*CDP*). Next, from the target-species proteins that possess both known and potential domains, we build a list of (known-potential) domain pairs 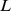, by randomly associating each new domain with one of the already known domains of the same protein. We let 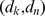 denote a pair of (known,potential) domains of 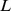. The list 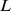 is used to estimate the FDR of 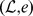. We assume that the proportion of false positives among the new domains 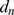 of 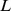 is generally the same as in all domains of 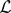. In particular, this assumes that, for a given e-value threshold, the proportion of false positives in domains of multi-domain proteins (those that are in 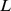) is the same as in domains of mono-domain proteins (that are not in 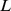). Although domains of mono- and multi-domain proteins are usually different, they generally share the same amino-acid composition, and there is no reason to believe that HMMs are more prone to false positives for either type.

Let 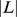 be the number of pairs in 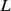. Now, we let 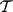 denote the probability that a pair in 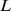 belongs to the set of CDPs 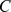, given that the potential domain is a true positive. Similarly, 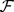 is the probability that a pair in 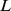 belongs to 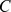, given that the potential domain is a false positive. We can express the expected number of pairs in 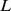 that belong to 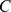 as 
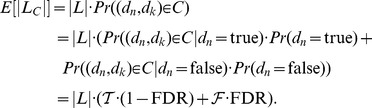



Thus, we have 
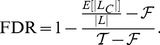
(1)



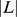
 is known, and 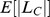 is estimated by the observed number of pairs in 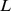 that belong to 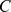. For 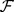, a list 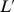 is created by randomly permuting new domains of the pairs in 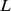. This is equivalent to randomly permuting the new domains in the proteins of the target species, and thus simulates a situation where almost all new domains are likely false positives. 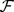 is estimated by the proportion of 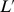 pairs that are in 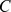. The procedure is repeated several times and averaged to obtain a better estimate. For 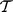, we use the known domain occurrences. A list 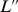 is created from (known,known) domain pairs observed in proteins with at least two known domains. This simulates the situation where all new domains are true positives, and 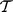 is estimated by the proportion of 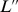 pairs that are in 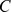.


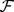
 and 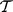 have very different estimated values. The value of 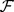 lies between 1% and 2%, independent of the method and E-value threshold. Hence, we used the value 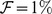 in Figure.2 For 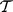, we generated several lists of (known,known) domain pairs and observed that the estimated 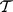 lies between 96% and 99%. Although these two values are relatively close, they may lead to different FDR estimates, especially for low FDRs. Thus, we provide two FDR estimates in our experiments: one computed with 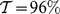 and one with 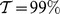.

Moreover, we used a bootstrap procedure [55] to measure the standard error of the FDR estimates. A bootstrapped list 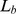 is built by randomly sampling with replacement 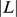 pairs of 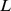. From this list, we compute a new FDR estimate 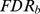 using the procedure described above, and the entire procedure is repeated a large number of times 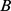 (here 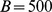). We then have a sample of 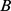 independent bootstrap replications of the FDR estimate, and we use the standard deviation of this sample as an estimate of the standard error. In Figure 2, this error is computed both for the 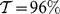 and 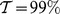 FDR estimates and is represented with dashed lines.

### Co-Occurrence Domain Discovery

CODD is a computational approach which enables us to select the most likely occurrences among a set of potential domain occurrences (with possibly numerous false positives), while controlling the false discovery rates associated with the predictions [56]. CODD utilises the same co-occurrence tendency used in the FDR estimation method described above but for a different purpose. Namely, given a set of new domain occurrences, CODD selects those that form, together with another domain of the same protein, a pair previously identified as being conditionally dependent (*i.e.* a pair of the CDP set). The domains selected this way are said to be *certified*. The certification can be done on the basis of the already known Pfam domains of the protein, but also on the basis of the other known InterPro (non Pfam) domains, or even on the basis of the other potential Pfam domains of the protein.

CODD provides an estimate of the FDR associated with the certified domains [56]. To this end, CODD estimates the probability of certifying a potential domain under the null hypothesis 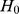 that it has been randomly predicted. This is done through computer simulations by shuffling the potential domains of all proteins. This creates a situation where the potential domains are independent of the validating domains, while preserving the domain distribution and the number of validating and potential domains in each protein. The certification procedure is applied to the shuffled domains, and the number of random domains certified is computed. The entire procedure is resumed several times (typically 1000 times) to get a reliable estimate of the expected number of domains our procedure would certify under the hypothesis that all potential domains are random. This number is then used to compute an estimate of FDR of the certification process with the formula 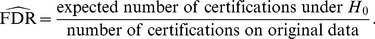
(2)


This approach is similar to that proposed in [57] to control the FDR associated with the multiple testing of several hypotheses.

## Supporting Information

Figure S1 **Sensitivity and accuracy of HHPRED+CODD and HMMER+CODD using the known Interpro domain occurrences for certifications.** This figure reports the number of new domains (x-axis) certified by HHPRED+CODD (in orange and green for the phylum specific and non-specific approaches, respectively) and HMMER+CODD (blue) using local (left) and global (right) alignments for various FDR thresholds (y-axis).
(PDF)

Figure S2 **Sensitivity and accuracy of HHPRED+CODD and HMMER+CODD using the potential domain occurrences for certifications.** This figure reports the number of new domains (x-axis) certified by HHPRED+CODD (in orange and green for the phylum specific and non-specific approaches, respectively) and HMMER+CODD (blue) using local (left) and global (right) alignments for various FDR thresholds (y-axis).
(PDF)

Table S1 **List of considered associations between domains and GO terms.**
(XLS)

Table S2 **List of the 36** ***P. falciparum*** **domain predictions that were manually analysed.**
(XLS)

Table S3 **List of the 37** ***L. major*** **domain predictions that were manually analysed.**
(XLS)

Table S4 **List of all predicted domains in** ***P. falciparum*** **.**
(XLS)

Table S5 **List of all predicted domains in** ***L. major*** **.**
(XLS)
