# Supplementary figures and images for: Identification of Divergent Protein Domains by Combining HMM-HMM Comparisons and Co-Occurrence Detection
Source: PLoS One. 2014 Jun 5;9(6):e95275. doi: 10.1371/journal.pone.0095275 (PMC4046975; doi:10.1371/journal.pone.0095275)

*P. falciparum*

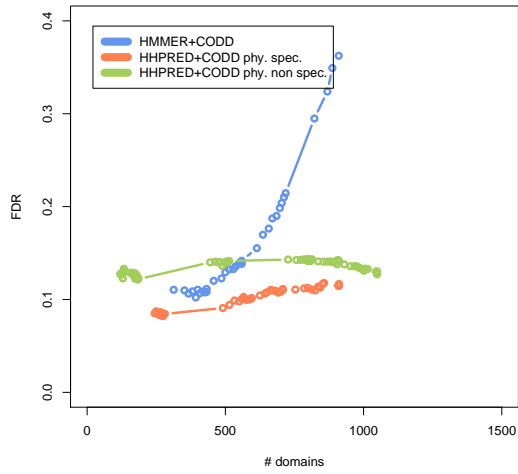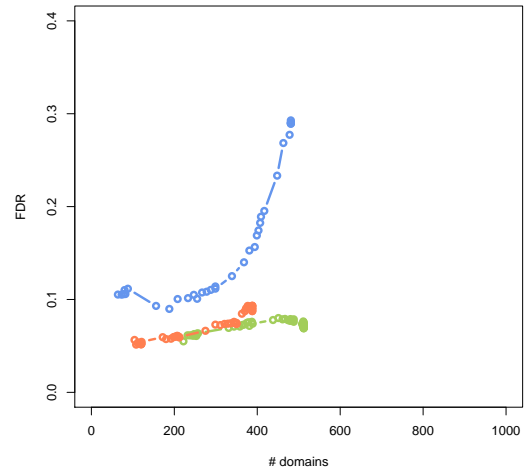

*L. major*

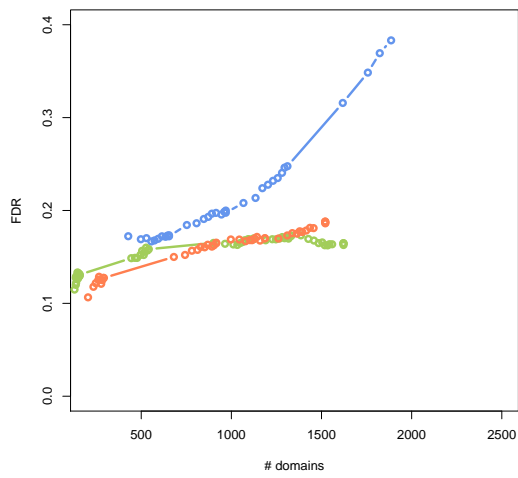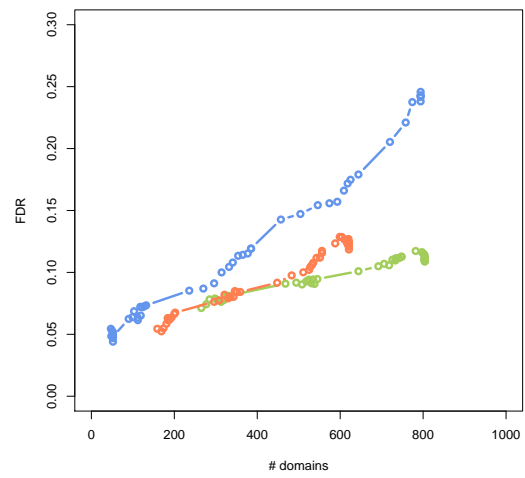

Supplement: Figure S1 — Sensitivity and accuracy of HHPRED+CODD and HMMER+CODD using the known Interpro domain occurrences for certifications. This figure reports the number of new domains (x-axis) certified by HHPRED+CODD (in orange and green for the phylum specific and non-specific approaches, respectively) and HMMER+CODD (blue) using local (left) and global (right) alignments for various FDR thresholds (y-axis). (PDF) [file pone.0095275.s001.pdf]

*P. falciparum*

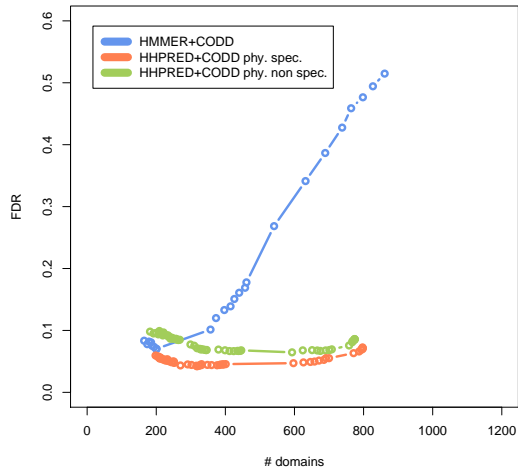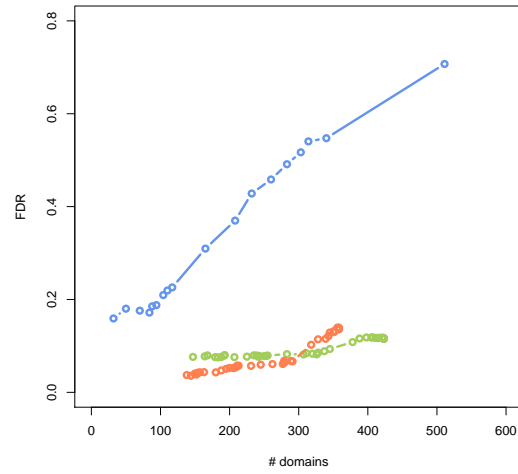

*L. major*

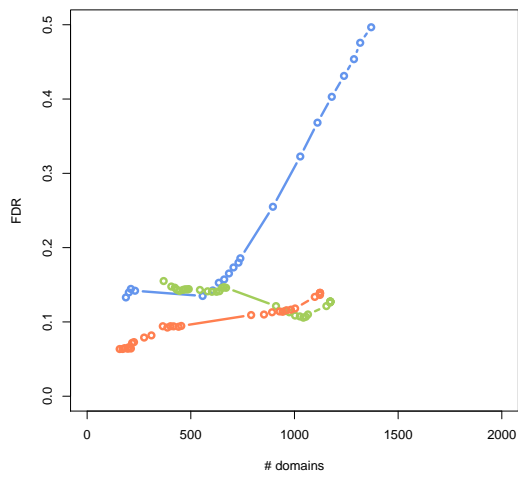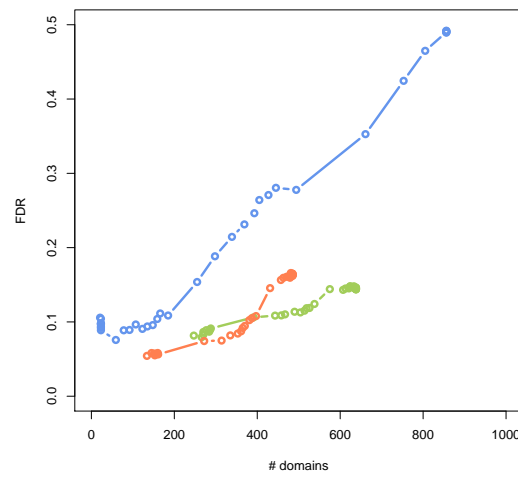

Supplement: Figure S2 — Sensitivity and accuracy of HHPRED+CODD and HMMER+CODD using the potential domain occurrences for certifications. This figure reports the number of new domains (x-axis) certified by HHPRED+CODD (in orange and green for the phylum specific and non-specific approaches, respectively) and HMMER+CODD (blue) using local (left) and global (right) alignments for various FDR thresholds (y-axis). (PDF) [file pone.0095275.s002.pdf]
